# The Possible Connection of Two Dual Function Processes: The Relationship of Ferroptosis and the JNK Pathway

**DOI:** 10.3390/ijms231911004

**Published:** 2022-09-20

**Authors:** Dóra Varga, Péter Hajdinák, Kinga Makk-Merczel, András Szarka

**Affiliations:** 1 Laboratory of Biochemistry and Molecular Biology, Department of Applied Biotechnology and Food Science, Budapest University of Technology and Economics, Szent Gellért tér 4, H-1111 Budapest, Hungary; 2 Biotechnology Model Laboratory, Faculty of Chemical Technology and Biotechnology, Budapest University of Technology and Economics, Szent Gellért tér 4, H-1111 Budapest, Hungary

**Keywords:** ferroptosis, JNK pathway, reactive oxygen species, cell death, cancer

## Abstract

Ferroptosis represents a typical process that has dual functions in cell fate decisions since the reduction and/or inhibition of ferroptosis is desirable for the therapies of diseases such as neurological disorders, localized ischemia-reperfusion, kidney injury, and hematological diseases, while the enhanced ferroptosis of cancer cells may benefit patients with cancer. The JNK pathway also has a real dual function in the fate of cells. Multiple factors suggest a potential link between the ferroptotic and JNK pathways; (i) both processes are ROS mediated; (ii) both can be inhibited by lipid peroxide scavengers; (iii) RAS mutations may play a role in the initiation of both pathways. We aimed to investigate the possible link between ferroptosis and the JNK pathway. Interestingly, JNK inhibitor co-treatment could enhance the cancer cytotoxic effect of the ferroptosis inducers in NRAS and KRAS mutation-harboring cells (HT-1080 and MIA PaCa-2). Since cancer’s cytotoxic effect from the JNK inhibitors could only be suspended by the ferroptosis inhibitors, and that sole JNK-inhibitor treatment did not affect cell viability, it seems that the JNK inhibitors “just” amplify the effect of the ferroptosis inducers. This cancer cell death amplifying effect of the JNK inhibitors could not be observed in other oxidative stress-driven cell deaths. Hence, it seems it is specific to ferroptosis. Finally, our results suggest that GSH content/depletion could be an important candidate for switching the anti-cancer effect of JNK inhibitors.

## 1. Introduction

The dual functions and the role of iron, ascorbate, and reactive oxygen species in cell fate decisions have been reviewed by our group [1] in the same special issue on “Dual Function Molecules and Processes in Cell Fate Decision 2.0”. In fact, the circle of molecules with dual functions is broadened by the present study.

According to the general approach in anti-cancer therapies, a special feature or factor is searched that distinguishes cancer cells from normal ones. Thus, the finding that the addition of a group of chemical compounds, such as erastin [2] and RSL-3 [3]-induced cell death (in tumor cells harboring RAS mutation) (inter alia in NRAS mutant HT-1080 cells) has received special interest. Later, it was found that erastin treatment caused a marked depletion in the glutathione (GSH) content of the cells, and RSL-3 behaved as a specific inhibitor for glutathione peroxidase 4 (GPX4). As a result of the low GSH level or the inhibition of GPX4, the levels of lipid hydroperoxides increased [4]. The morphologic, biochemical, and genetic characteristic features of the observed cell death due to erastin or RSL-3 treatments were totally different from any other known cell death types, such as apoptosis, necrosis, and autophagy [4]. Furthermore, this could be inhibited by an iron chelator (deferoxamine) or by the antioxidant, vitamin E, confirming that this form of cell death is related to intracellular iron and reactive oxygen species (ROS) [3,4]. Due to its strong iron dependency, this novel form of cell death was called ferroptosis [4]. Later, it was proposed that autophagy is involved in ferroptotic cell death [5]. As was recently revealed, ferroptosis is not unique to cancer cells; its role was also proposed in different neurological diseases [6], such as stroke [7], Alzheimer’s disease [8], Parkinson’s disease [9], Huntington’s disease [10], and multiple sclerosis [11], and also in localized ischemia-reperfusion injury, kidney injury and hematological diseases [12]. In addition, our research group described its role in acetaminophen-induced hepatotoxicity [13]. Ferroptosis is a typical instance of a process with dual functions in cell fate decision since the reduction and/or inhibition of ferroptosis is desirable for the therapies of the above-listed diseases, while the enhanced ferroptosis of cancer cells may benefit patients with cancer [1]. Indeed, persistent oxidative stress is a common feature of cancer cells, as is evidenced by elevated oxidative DNA lesions [14]. It has been proposed that cancer cells are able to adjust oxidative stress to a level which is sufficient enough to maintain their survival but not to initiate their elimination. Necessarily, this adaptation of cancer cells to elevated ROS levels treads a very fine line between their proliferation and cell death. Therefore, increased ROS levels are frequently used as cytotoxins in cancer patients [14]. In this way, ferroptosis, the only unequivocally oxidative stress and lipid peroxidation-driven cell death, definitely gives a novel perspective to anti-cancer therapy.

The JNK pathway can be activated by a variety of cellular stressors, such as the exposure of cells to ultraviolet light, heavy metals, irradiation, chemotherapeutic drugs, and the aforementioned reactive oxygen species. Thus, it is also sometimes referred to as the stress-activated protein kinase pathway [15]. It is important to note that JNK signaling can also be activated by the activation of RAS. RAS activation results in Rac activation, and Rac can induce the activation of JNK via MLK. The downstream targets of the JNKs include c-Jun, ATF-2, ELK2, and NRF2 [15].

It has been well documented that ROS are also potent inducers of JNK [16]. In the absence of any oxidative stress, reduced thioredoxin binds to the N-terminal of ASK1, an upstream regulator of JNK, preventing its activation. ROS induce the oxidation of thioredoxin. The oxidized thioredoxin dissociates from ASK1, allowing ASK1 to oligomerize, autophosphorylate, and become activated. The activated ASK1 activates MKK4/7, and then these, in turn, activate JNK [15]. The role of ROS in the activation of the JNK pathway was underlined by antioxidants since superoxide anion- and lipid peroxide-scavengers could inhibit the activation of JNK [17].

The outcome of JNK or p38 activation strongly depends on the cell type and the cellular context. Thus, it is a process with a real dual function towards the fate of cells. Albeit, many JNK-activating stimuli are proapoptotic, it was reported that c-Jun-deficient fibroblasts are more sensitive to UV-induced apoptosis, suggesting that c-Jun could also act as an antiapoptotic factor [18].

Multiple factors suggest a potential link between the ferroptotic and JNK pathways; (i) both processes are ROS mediated; (ii) both can be inhibited by lipid peroxide-scavengers; (iii) RAS mutations may play a role in the initiation of both pathways. In the present study, we aim to elucidate the link between ferroptosis and the JNK pathway.

## 2. Results

**Figure 1:**
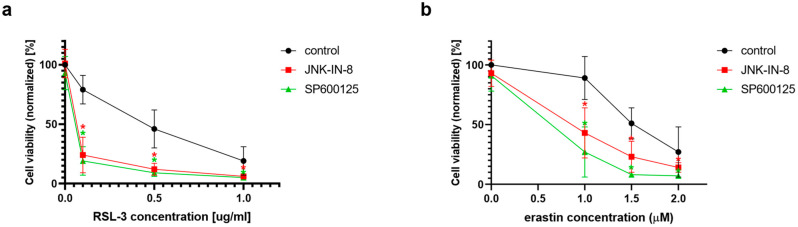
Pharmacological inhibition of JNK activity enhances the effect of ferroptosis inductors in HT-1080 cells. HT-1080 cells were treated on a 96-well plate for 24 h with the ferroptosis-inducing compounds (**a**) RSL-3 (0.1–1 µg/mL) or (**b**) erastin (1–2 µM). RSL-3 treatment, in all three applied concentrations, and 1.5 µM and 2 µM erastin treatment, significantly decreased cell viability. Co-treatment with JNK inhibitors, JNK-IN-8 (10 µM, the first irreversible JNK inhibitor) or SP600125 (5 µM, a frequently used reversible, ATP-competitive JNK inhibitor) significantly decreased cell viability compared to only RSL-3 or erastin treatment. Cell viability was measured by MTT assay. Data are normalized to an untreated control, and each data point represents the average ± SD from at least three independent experiments. * Significantly different (*p* < 0.05) from group control (RSL-3 or erastin treated without co-treatment).

Since the first comprehensive description of ferroptosis occurred for HT-1080 cells [4], and this cell line is the object of the majority of the studies on ferroptosis, this cell line was chosen as the object of the investigation for the potential connection between ferroptosis and the JNK pathway. In the first set of experiments, ferroptosis was induced by the two typical ferroptosis inducers: erastin and RSL-3. As expected, both erastin and RSL-3 treatments resulted in a significant decrease in cell viability (Figure 1). Interestingly, the co-treatment of HT-1080 cells with JNK inhibitors, such as JNK-IN-8 or SP600125, could decrease cell viability even more when compared to the cells treated solely with RSL-3 or erastin (Figure 1).

**Figure 2:**
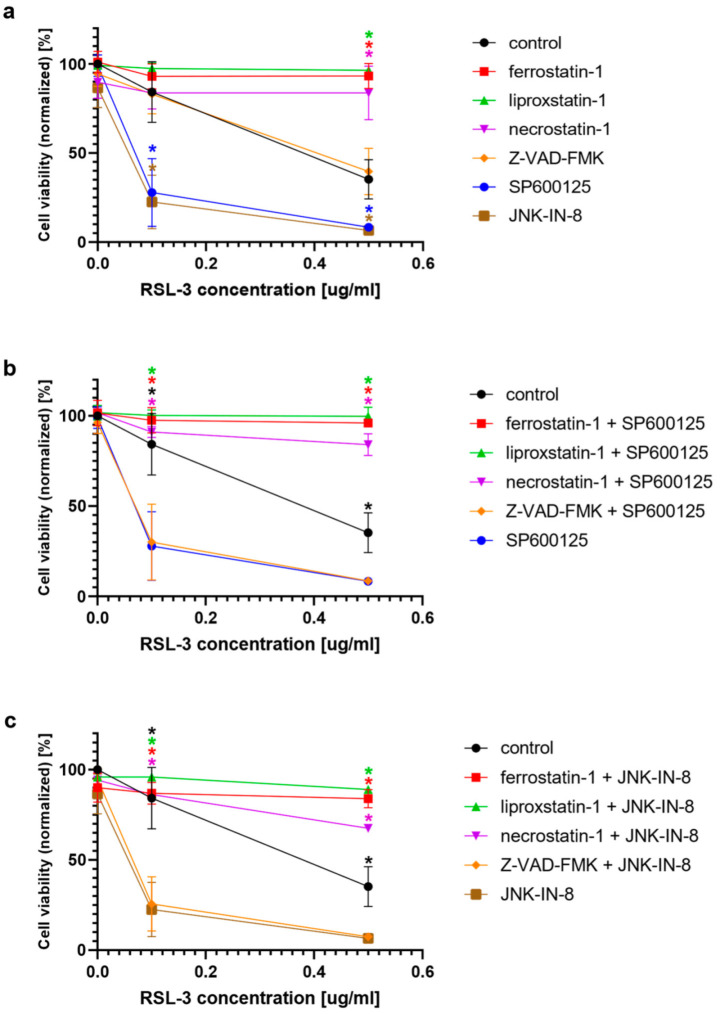
The potential effect of the inhibitors in alleviating cell death induced by RSL-3 in the presence or absence of the JNK inhibitors. HT-1080 cells were treated on a 96-well plate for 24 h with the ferroptosis-inducing compound RSL-3 (0.1 µg/mL and 0.5 µg/mL) in the presence or absence of inhibitors of interest and combinations of them: ferrostatin-1 (5 µM), liproxstatin-1 (500 nM), necrostatin-1 (50 µg), Z-VAD-FMK (50 µg), JNK-IN-8 (10 µg) and SP600125 (5 µg). Ferroptosis inhibitors, ferrostatin-1, liproxstatin-1, and necroptosis inhibitor necrostatin-1, inhibit RSL-3 induced ferroptosis both in the presence or absence of the JNK inhibitors, while the apoptosis inhibitor, Z-VAD-FMK, has no effect in either case. Cell viability was measured by MTT assay. Data are normalized to the untreated control, and each data point represents the average ± SD from at least three independent experiments. (**a**) * Significantly different (*p* < 0.05) from group control (RSL-3 treated without co-treatment). (**b**) * Significantly different (*p* < 0.05) from RSL-3 and JNK-IN-8 co-treated. (**c**) * significantly different (*p* < 0.05) from RSL-3 and SP600125 co-treated.

**Figure 3:**
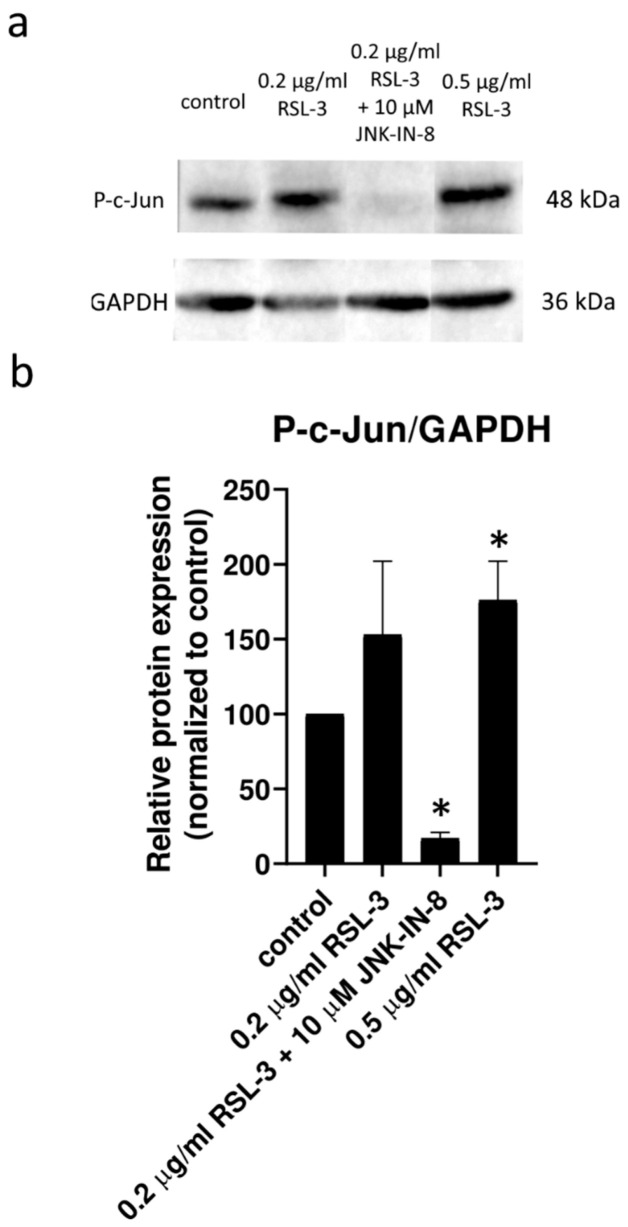
Western blot analysis of total protein samples for c-Jun phosphorylation in HT-1080 cells in response to RSL-3 treatment. (**a**) Western blot analysis was performed as described below. Phosphorylated c-Jun protein levels were determined after 24 h treatment with RSL-3 (0.2 µg/mL or 0.5 µg/mL) in the presence or absence of the JNK inhibitor, JNK-IN-8 (10 µM). GAPDH were labeled for loading the control. (**b**) Densitometry data represent the intensity of phosphorylated c-Jun normalized to GAPDH; each data point represents the average ± SD from at least three independent experiments. * Significantly different (*p* < 0.05) from control.

**Figure 4:**
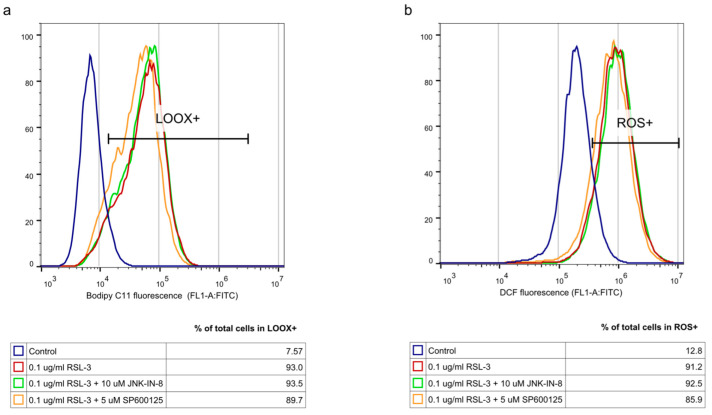
Pharmacological inhibition of JNK activity does not affect the oxidative aspects of RSL-3-induced ferroptosis. HT-1080 cells were treated on a 24-well plate for 2 h with the ferroptosis-inducing compound, RSL-3 (0.1 µg/mL), in the presence or absence of the JNK inhibitors, JNK-IN-8 (10 µM) or SP600125 (5 µM). RSL-3 treatment causes oxidative stress through (**a**) lipid peroxidation (LOOX) and (**b**) ROS formation, but JNK inhibition does not affect either lipid peroxidation or ROS formation. Cells were prepared, stained, and measured with a flow cytometer, as described above, using a Bodipy C11 fluorescent probe for lipid peroxidation and a DCF fluorescent probe for ROS measurement. Control samples were fluorescent-labelled but untreated. One representative experiment is shown. Cell population was gated using FSC-W for singlets and propidium iodide negative staining (living cells).

**Figure A1:**
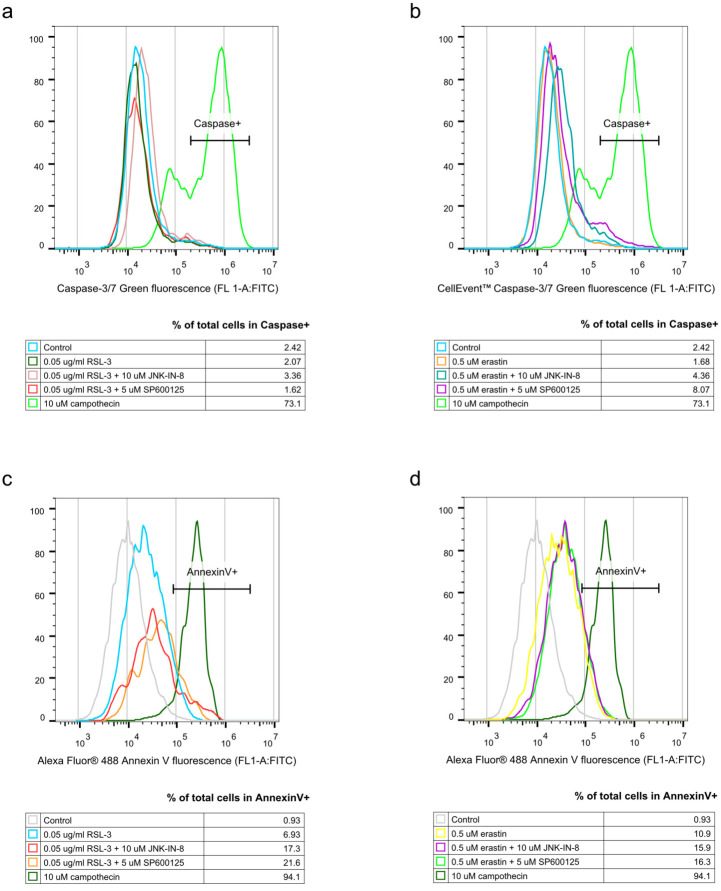
Measurement of apoptosis markers after treatment with ferroptosis inducers and JNK inhibitors. The HT-1080 cells were treated on a 6-well plate for 24 h with the ferroptosis inducing compounds (**a**,**c**) RSL-3 (0.05 µg/mL) or (**b**,**d**) erastin (0.5 µM) in the presence or absence of the JNK inhibitors, JNK-IN-8 (10 µM) or SP600125 (5 µM). Cells were prepared, stained, and measured with a flow cytometer as described above (**a**,**b**) using CellEvent™ Caspase-3/7 Green Detection Reagent for measuring caspase activation and (**c**,**d**) Annexin V Alexa Fluor 488 conjugate for the measurement of the externalization of phosphatidylserine on the plasma membrane. Control samples were fluorescent-labelled but untreated. One representative experiment is shown. Cell population was gated using FSC-W for singlets and propidium iodide negative staining (living cells).

In the second turn of our experiments, an attempt was made to elucidate the background of the increased rate of cell death due to JNK inhibitor co-treatment. Thus, we tried to suspend the effect of JNK inhibitors by adding inhibitors from different cell-death types (Figure 2). Furthermore, the effect of ferroptosis inducers on the activation of the JNK pathway was investigated by determining the amount of phosphorylated c-Jun using Western blot (Figure 3). On the basis of the observed cell viability, the JNK inhibitors could amplify the effect of the ferroptosis inducers (Figure 1 and Figure 2). The sole JNK-inhibitor treatment did not affect the cell viability (Figure 1 and Figure 2). Since the activation of the JNK pathway was associated with the induction of apoptosis [19], the effect of the pan-caspase inhibitor, Z-VAD-FMK, was also investigated, but it did not show any effect (Figure 2b,c). The direct caspase activity measurement and apoptosis detection (by measuring the externalization of phosphatidylserine on the plasma membrane using fluorescent-tagged annexin V) also reinforced these results (Appendix A, Figure A1). All these results suggest that the JNK inhibitors alone are not able to induce cell death in the HT-1080 cell line, but they amplify the anti-cancer effect of the ferroptosis inducers. Furthermore, it seems apoptosis is not involved in this cell death amplification. In addition, the activation of the JNK pathway was observed due to RSL-3 treatment, which was eliminated by the JNK inhibitor treatment (Figure 3). Since the elevation of the lipid peroxides occur due to the ferroptosis inducers being a key element of ferroptosis [4], we investigated whether the JNK inhibitors had any effect on it. Since ROS have high activity and a very short lifetime, elevated ROS levels can only be detectable in living cells for a short period of time. Thus, it is necessary to measure them after a shorter period of treatment (2 h). On the contrary, the development of elevated ROS levels (and the effect of this, such as cell viability (or even GSH depletion or changes in protein expression) takes longer; thus, 24 h treatments were applied in these cases. As expected, both the DCF fluorescent-detectable ROS levels and the Bodipy C11-detectable lipid peroxide levels, were significantly elevated due to RSL-3 treatment (Figure 4). However, neither of these were influenced by the JNK inhibitor co-treatment (Figure 4).

**Figure 5:**
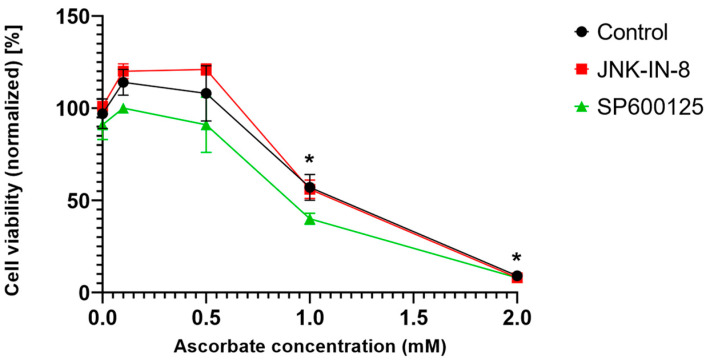
Pharmacological inhibition of JNK activity does not affect ascorbate-induced cell death in HT-1080 cells. HT-1080 cells were treated on a 96-well plate for 24 h with ascorbate (0.1–2 mM). Co-treatment with JNK inhibitors, JNK-IN-8 (10 µM) or SP600125 (5 µM), does not significantly affect the ascorbate-induced cell death. Cell viability was measured by MTT assay. Data are normalized to the untreated control and each data point represents the average ± SD from at least three independent experiments. * Significantly different (*p* < 0.05) from untreated.

Since ferroptosis is an oxidative stress- and lipid peroxidation-driven cell death, we investigated whether the effect of the JNK inhibitors is unique to ferroptosis or if it was observable in another oxidative stress-driven cell death. Since pharmacologic ascorbate-induced cell death and ferroptosis share common features, such as iron dependency, ROS production, lipid peroxidation, caspase independency, and the possible involvement of autophagy [20], we chose to test the aforementioned in question. Interestingly it was found that the JNK inhibitors could not amplify pharmacologic ascorbate-induced cell death (Figure 5). Hence, it seems the effect of the JNK inhibitors is specific to the ferroptosis inducers.

**Figure 6:**
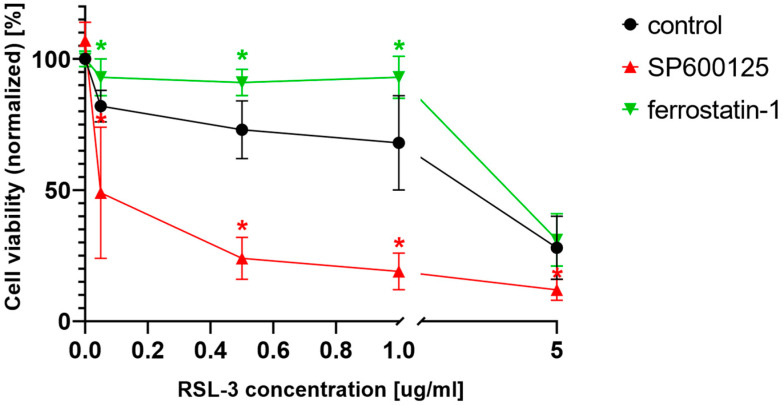
Pharmacological inhibition of JNK activity enhances the effect of ferroptosis inductor, RSL-3, in MIA PaCa-2 cells. MIA PaCa-2 cells were treated on a 96-well plate for 24 h with the ferroptosis-inducing compound, RSL-3 (0.05–5 µg/mL). RSL-3 treatment, within all applied concentrations, significantly decreased cell viability. Co-treatment with JNK inhibitor, SP600125 (5 µM), significantly decreased cell viability compared to only RSL-3 treatment. Ferroptosis inhibitor, ferrostatin-1 (10 µM), inhibits ferroptosis inducement at lower RSL-3 concentrations. Cell viability was measured by MTT assay. Data are normalized to untreated control and each data point represents the average ± SD from at least three independent experiments. * Significantly different (*p* < 0.05) from group control (RSL-3 treated without co-treatment).

As stated in the introduction, ferroptosis was primarily considered to be a specific cell death in RAS mutant cancers [2,3]. Furthermore, JNK signaling can also be activated by the activation of HRAS [21,22] and NRAS [23]. Thus, the potential ferroptosis amplifier effect of the JNK inhibitors was investigated on another RAS mutant cell line (MIA PaCa-2). This cell line, harboring KRAS mutation, behaved similarly to the NRAS mutant HT-1080 cells (Figure 6). Cell viability significantly decreased due to RSL-3 treatment (Figure 6) and was further decreased by the co-treatment with those cells with the JNK inhibitor SP600125. The specific ferroptosis inhibitor, ferrostatin-1, could inhibit the decrease of cell viability at lower RSL-3 concentrations (Figure 6).

**Figure 7:**
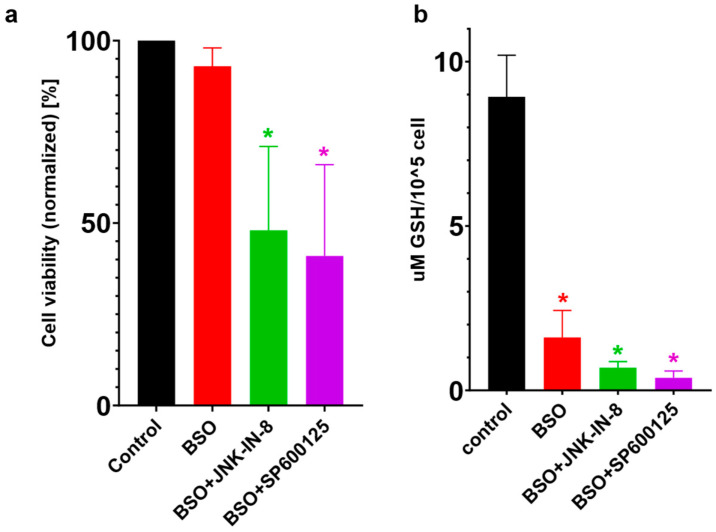
The effect of JNK inhibition within BSO-treated HT-1080 cells. HT-1080 cells were pre-treated on a 24-well plate for 24 h with a 100 μM final concentration of BSO. Then, the culture medium was further supplemented with the JNK inhibitors, JNK-IN-8 (10 µM) or SP600125 (5 µM). After another 24 h, cell viability was measured by (**a**) MTT assay, and (**b**) the GSH levels were measured with monochlorbimane (mClB) derivatization followed by HPLC separation. While BSO treatment significantly decreased the GSH levels, it did not affect the cell viability significantly. Co-treatment with the JNK inhibitors and BSO also significantly lowered the GSH levels, and this treatment also decreased the cell viability significantly. (**a**) Data are normalized to the untreated control, and each data point represents the average ± SD from at least three independent experiments. * Significantly different (*p* < 0.05) from untreated. (**b**) Each data point represents the average ± SD from at least three independent experiments. * Significantly different (*p* < 0.05) from untreated.

GSH, more precisely, GSH depletion, plays a central role in the induction of ferroptosis since the specific ferroptosis inducer, erastin, causes a marked depletion in GSH [24]. Furthermore, GSH is an indispensable substrate of the lipid ROS scavenger enzyme, GPX4, which plays a central role in ferroptosis since its specific inhibition by RSL-3 causes ferroptotic cell death [24]. Thus, GSH content/depletion could be another candidate for the switching the anti-cancer effect of the JNK inhibitors. Therefore, the effect of JNK inhibitor co-treatment was investigated in BSO-treated (GSH depleted) HT-1080 cells. It was found that, similar to the erastin treatment (Figure 1b), cell viability could be further decreased by JNK inhibitor co-treatment with GSH depleted (due to BSO treatment) HT-1080 cells (Figure 7). It seems that GSH may play a central role in the cell death amplifying effect of the JNK inhibitors.

## 3. Discussion

Since cancer cells seem to exist under persistent oxidative stress, and extra oxidative pressure can easily lead to their death, increased ROS levels are frequently used as cytotoxins in cancer patients [14]. Hence, the inductors of ferroptosis, the only unequivocally oxidative stress- and lipid peroxidation-driven cell death, may give a novel perspective on anti-cancer therapy. Not accidentally, the role of ferroptosis in cancer treatment is a matter of intensive research [25]. This is why our research group found it surprising that the connection between this lipid ROS-driven cell death and one of the major oxidative stress-triggered signaling pathways, the JNK pathway, has not yet been investigated. There are several other linking points, beyond oxidative stress, between ferroptosis and the JNK pathway, such as their potential links to the RAS pathway and their relationship to lipid peroxide scavengers or GSH [15,16,17,18].

Fortunately, at the beginning of our experiments, it could be proven that there is a link between these two oxidative stress-driven pathways. The viability of ferroptosis inducer (erastin or RSL-3)-treated HT-1080 fibrosarcoma cell line–which was used in the description of ferroptosis [4]–could be further reduced by co-treatment with JNK inhibitors (JNK-IN-8 or SP600125) (Figure 1). Since the JNK inhibitors alone did not have any effect on cell viability (Figure 2a), and this cancer cytotoxic effect of the JNK inhibitors could only be suspended by ferroptosis inhibitors (Figure 2b), it seems that the JNK inhibitors “just” amplify the effect of the ferroptosis inducers. Although the receptor-interacting protein kinase 1 (necroptosis) inhibitor, necrostatin-1, behaved similarly to the ferroptosis inhibitor, ferrostatin-1 (Figure 2b,c), it can still be easily discussed due to the tight connection between necroptosis and ferroptosis. Furthermore, this phenomenon has already been observed previously by our own and independent research groups [13,26]. Using the pan-caspase inhibitor, Z-VAD-FMK (Figure 2b,c), and by determining caspase activation, the involvement of the caspases was ruled out regarding the reduction of cell viability induced by the JNK inhibitors. All these results suggest that the JNK inhibitors are not able to induce cell death in the HT-1080 line. However, they amplify the anti-cancer effect of the ferroptosis inducers, and apoptosis is not involved in this amplification.

At this point, we have to take a step back and think over the dual role of the JNK pathway in the fate of cells. From the perspective of ferroptosis, it is more than important that JNK can not only be activated by ROS but can also be oxidized from the byproducts of ROS generation. In particular, the lipid peroxidation product, 4-hydroxynonenal, has been shown to activate JNK [27,28]. The fragmentation of lipid alkoxyl radicals–generated during the induction of ferroptosis–yields the production of reactive aldehydes, such as malondialdehyde, 4-hydroxynonenal (HNE), and acrolein [29]. Cells that overexpress the members of the aldo-keto reductase type 1C family, which have the ability to detoxify the toxic lipid metabolites produced by the oxidative lipid fragmentation during the execution of ferroptosis, showed resistance to the ferroptosis inducer erastin [30]. The potential role of the lipid alkoxyl radical derivative, acrolein, in plant ferroptosis, such as cell death, has also been shown by our research group [31]. Thus, it has particular importance that 4-hydroxynonenal is an activator of both the ferroptotic and JNK pathways. Not surprisingly, the prolonged activation of the JNK pathway triggers cell death in hepatocytes [32,33,34,35,36], and the inhibition of c-Jun function could decrease this hepatocyte cell death [32]. All these observations are in concordance with our results that the amount of phosphorylated c-Jun was double that of the control due to RSL3 treatments. This elevated level of phosphorylated c-Jun could be diminished by JNK inhibitor treatment (Figure 3).

Following this train of thought, the elevation of cell viability should have been observed due to JNK inhibitor treatment. However, it has even occurred in an opposing way. Here, it should be emphasized that the outcome of JNK or p38 activation strongly depends on the cell type and the cellular context [18]. Recently it was demonstrated in vitro that JNK is required for the maintenance of the self-renewal and tumor-initiating capacity of human glioblastoma and pancreatic cancer stem cells (CSCs) or cancer stem-like cells (CSLCs) [37]. Furthermore, using preclinical animal models, it was shown that systemic JNK inhibitor administration in tumor-bearing mice eliminates the cell population within the tumors effectively and safely [37]. These data suggest that JNK could serve as a therapeutic target for cancers whose CSC/CSLCs are dependent on JNK [37]. Although JNK was initially identified as an HRAS-activated kinase [21,22], it was later shown that mutated KRAS activates JNK both in vitro and in vivo [38,39]. It is worth noting that the KRAS G12D mutation, the most common mutant allele in pancreatic cancers [40], has been shown to preferentially activate the JNK pathway [38]. It seems that the KRAS activation of JNK has a critical role in the maintenance of the self-renewal and tumor-initiating capacity of pancreatic CSCs/CSLCs. Thus, it is more than important that siRNAs directed against KRAS cause the reduced phosphorylation of JNK and c-Jun, suggesting that KRAS expression is required for the maintenance of JNK signaling in PANC-1 CSLCs [37]. All these results suggest that the KRAS–JNK axis could be a potential target in CSC/CSLC-directed therapies against pancreatic cancer. Importantly, similar results have been observed in the case of HT-1080 human fibrosarcomas and DLD-1 colon carcinoma cells. Mutated RAS was important for the growth transformation of both cell lines. The activation of JNK definitely correlated with the expression of oncogenic RAS in HT-1080 cells [23]. All these observations, again, underline the importance of our results: that JNK inhibitors can further deepen cancer cell toxicity due to ferroptosis inducer treatment (Figure 1 and Figure 2). Since JNK-regulated pathways have shown that they play a crucial role in both cell proliferation and cell death [41], they can be called typical dual function processes in cell fate decisions.

To clarify the background of the cell death amplification effect of the JNK inhibitors, we investigated whether the JNK inhibitors have any effect on the elevation of ROS and lipid peroxides, which have a critical role in the initiation of ferroptosis [4]. RSL-3 treatment resulted in significantly elevated DCF fluorescent-detectable ROS levels and Bodipy C11-detectable lipid peroxide levels. However, neither of these was influenced by the JNK inhibitor co-treatment (Figure 4). It should be noted that recent studies have highlighted the involvement of JNK in the activation of NRF2. The activated NRF2 was released from KEAP1 and translocated to the nucleus under the control of the ERK- and JNK-signaling pathways [42]. In this way, it is possible that the inhibition of JNK resulted in the inactivation of NRF2. Since NRF2 induces the expression of genes whose products are involved in the detoxification of reactive oxygen species, it probably counteracts the ferroptotic pathway. Furthermore, NRF2 can inhibit ferroptosis by altering the expression of multiple genes in the regulation of labile iron, the synthesis of GSH and lipid hydroperoxides reduction by GPX4, and the FSP1-dependent CoQ system [43]. All of these observations may suggest that the inhibition of JNK by JNK-IN-8 or SP600125 might have caused the inhibition of NRF2 too and maintained the elevated ROS and lipid ROS levels due to longer exposure to the ferroptosis inducers, sensitizing the HT-1080 to ferroptosis and deepening its cancer cell toxicity (Figure 1 and Figure 2).

Since ferroptosis is an oxidative stress- and lipid peroxidation-driven cell death, we investigated whether the effect of the JNK inhibitors is unique to ferroptosis or if it can be observed in other oxidative stress-driven cell deaths. Pharmacologic ascorbate-induced cell death was chosen to test the aforementioned question since it shares common features with ferroptosis, such as iron dependency, production of ROS, lipid peroxidation, caspase independence, and the possibility of autophagy involvement [20]. Interestingly, it was found that the JNK inhibitors could not amplify the pharmacologic ascorbate-induced cell death (Figure 5). Hence, it seems the cancer cell death amplifying effect of the JNK inhibitors is specific to the ferroptosis inducers and is not necessarily a common feature in oxidative stress-driven cell deaths.

As it was stated earlier, JNK was originally identified as an HRAS-activated kinase [21,22]; however, later, it was shown that mutated KRAS also activates JNK both in vitro and in vivo [38,39], and the activation of JNK definitely correlated with the expression of oncogenic NRAS in HT-1080 cells [23]. Thus, after investigating the effects of the JNK inhibitors on the NRAS mutant HT-1080 cell line, we continued our experiments to elucidate their effect on a cell line which harbors a KRAS mutation. The chosen KRAS mutant cell line, MIA PaCa-2, behaved very similarly to the NRAS mutant HT-1080 cells since their cell viability significantly decreased due to RSL-3 treatment (Figure 6) and was further decreased by co-treatment with the JNK inhibitor, SP600125 (Figure 6). Furthermore, the specific ferroptosis inhibitor, ferrostatin-1, similarly to the HT-1080 cell line, could inhibit the decrease of cell viability at lower RSL-3 concentrations (Figure 6). These observations suggest a general cytotoxic deepening effect from the JNK inhibitors on ferroptosis inducer-treated RAS mutant cancer cell lines.

From the beginning of ferroptosis research, it has been well known that GSH depletion plays a central role in the induction of ferroptosis since RSL-3 behaves as a specific inhibitor for the lipid ROS scavenger, GPX4 [24], and erastin causes a marked depletion in GSH, the substrate of GPX4 [4,24,26]. Later, it was observed that the HNE-induced phosphorylation of JNK/c-Jun could be completely blocked by GSH supplementation [32], demonstrating that JNK/c-Jun overactivation resulted from GSH depletion. GSH supplementation blocked JNK/c-Jun activation and cell death, demonstrating that JNK/c-Jun overactivation is the downstream effect of GSH depletion. Further results also reinforced this assumption since the inhibition of GSH depletion resulted in decreased HNE-induced JNK activation [32]. Therefore, the effect of JNK inhibitor co-treatment was also investigated in BSO-treated (GSH depleted) HT-1080 cells. It was found that, similar to erastin treatment (Figure 1b), cell viability could be further decreased by JNK inhibitor co-treatment in BSO-treated HT-1080 cells (Figure 7). Based on these observations, GSH may play a central role in the cell death amplifying effect of JNK inhibitors. Thus, GSH content/depletion could be another candidate for switching the anti-cancer effect in JNK inhibitors.

In conclusion, JNK inhibitor co-treatment can enhance the cancer cytotoxic effect of ferroptosis inducers in NRAS and KRAS mutation-harboring cells (HT-1080 and MIA PaCa-2). The sole JNK inhibitor treatment did not affect cell viability. Furthermore, the cancer cytotoxic effect of the JNK inhibitors could only be suspended by ferroptosis inhibitors (Figure 2b); thus, it seems that JNK inhibitors “just” amplify the effect of the ferroptosis inducers. This cancer cell death amplifying effect of the JNK inhibitors could not be observed in other oxidative stress-driven cell deaths. Hence, it seems that it is specific to ferroptosis. The possible inhibition of the NRF2 pathway by the JNK inhibitors might sensitize the HT-1080 cells to ferroptosis and might contribute to enhanced cancer cell toxicity. As GSH content/depletion could provide a candidate for switching the anti-cancer effect of the JNK inhibitors, it seems that GSH could play a central role in the cell death amplifying effect of the JNK inhibitors. Necessarily, further experimental evidence is needed to reinforce these assumptions.

## 4. Materials and Methods

### 4.1. Cell Cultures

The HT-1080 (obtained from ECACC Database, Catalogue No.: 85111505) fibrosarcoma cell line and MIA PaCa-2 (obtained from ATCC Database, ATCC Number: CCL-121), a human pancreatic cancer cell line used in the experiments, were cultured according to ATCC guidelines. Cells were grown in a cell culture incubator (Thermo Scientific, Waltham, MA, USA Forma™ Series II 3111) at 37 °C, 5% CO_2_, 100% relative humidity. The complete culture medium for the HT-1080 cell line comprised of high glucose DMEM with stable glutamine (Thermo Fisher Scientific, Gibco™, 10566016 or Sigma-Aldrich^®^, St. Louis, MO, USA, D0819) supplemented with 10% FBS (Sigma-Aldrich^®^, F7524), 1% antibiotic/antimycotic (Sigma-Aldrich^®^, A5955). HT-1080 cells were subcultured routinely before reaching 100% confluence in a ratio of 1:4 or 1:6. The complete culture medium for the MIA PaCa-2 cell line comprised of high glucose DMEM with stable glutamine (Gibco™, 10566016 or Sigma-Aldrich^®^, D0819) supplemented with 10% FBS (Sigma-Aldrich^®^, F7524) and 2.5% horse serum (Gibco™, 16050130). MIA PaCa-2 cells were subcultured routinely before reaching 100% confluence in a ratio of 1:5 or 1:6.

### 4.2. Treatment of HT-1080 and MIA PaCa-2 Cells for MTT Viability Assay, Flow Cytometry or GSH Measurement

The HT-1080 cells were seeded homogenously 24 h prior to treatment in either 96-, 24-, or 6-well plates (Thermo Scientific™ BioLite) at a seeding density of 1.5 × 10^4^, 1 × 10^5^, 5 × 10^5^ cells, respectively, in complete culture medium. MIA PaCa-2 cells were seeded homogenously 24 h prior to treatment in 96-well plates (Thermo Scientific™ BioLite) at a seeding density of 3 × 10^4^. After 24 h of incubation, the complete culture medium was renewed and supplemented with various compounds for treatment.

For ferroptosis induction, cells were treated with 0.05–5 μg/mL RSL-3 (MCE^®^, Monmouth Junction, NJ, USA, HY-100218A, solved in DMSO) or 0.5–10 μM erastin (MCE^®^, HY-15763, solved in DMSO). For inhibitor profile studies, the RSL-3 or erastin supplemented complete growth medium was further supplemented by one or two of the following: 5 μM final concentration of SP600125 (MCE^®^, HY-12041, solved in DMSO) for HT-1080 cells, 10 μM final concentration for MIA PaCa-2 cells, 10 μM final concentration of JNK-IN-8 (MCE^®^, HY-13319, solved in DMSO), 50 μM final concentration of Z-VAD-FMK (MCE^®^, HY-16658B, solved in DMSO), 50 μM final concentration of necrostatin-1 (Santa Cruz Biotechnology, Dallas, TX, USA, sc-200142, solved in DMSO), 500 nM final concentration of liproxstatin-1 (MCE^®^, HY-12726, solved in DMSO), 5 μM final concentration of ferrostatin-1 (MCE^®^, HY-100579, solved in DMSO) for HT-1080 cells, 10 μM final concentration for MIA PaCa-2 cells.

For the measurement of apoptosis markers, camptothecin (Selleckchem, Houston, TX, USA, S1288, solved in DMSO) was used as a positive control in 5–10 μM final concentrations.

To investigate the effect of JNK inhibition on ascorbate induced cell death in HT-1080 cells, 100 mM ascorbic acid (Sigma-Aldrich^®^, A4544) stock solution was prepared before each treatment in complete culture medium and the pH was adjusted to phenol red neutral with sodium hydroxide. Cells were treated with 0.1–5 mM ascorbate supplemented culture medium with or without further supplemented 5 μM final concentration of SP600125, 10 μM final concentration of JNK-IN-8, or 5 μM final concentration of ferrostatin-1.

For L-Buthionine-sulfoximine (BSO, Sigma-Aldrich^®^, B2640) treatment, 100 mM BSO stock solution was prepared before each treatment in distilled water. The cells were seeded homogenously in 24-well plates in complete culture medium supplemented with BSO in 100 μM final concentration. After 24 h of incubation, the culture medium could be further supplemented with 5 μM final concentration of SP600125 or 10 μM final concentration of JNK-IN-8. After an additional 24 h of treatment time, cells were processed for either MTT assay or GSH measurement.

Solvent (DMSO) controls were used in all cases for inhibitor profile studies, max. DMSO content was 0.2 *v*/*v*%).

### 4.3. Measurement of Cell Viability with the MTT Assay

Cell viability for toxicity and inhibitor profile determination was measured in either 96- or 24-well plates. Cells were seeded and treated as described above. After 24 h of treatment, the medium was discarded, the plate was washed twice with PBS. To assess cell viability, complete growth medium supplemented with 1/10 volume 5 mg/mL MTT (Sigma-Aldrich^®^, M2003, dissolved in PBS) was added to the plate and incubated for 35 min until sufficient formazan crystals were present. After incubation, the medium was discarded and the formazan crystals were dissolved by adding DMSO and incubating the plate for further 10 min at 37 °C. The absorbance of the formazan solution was determined by a microplate spectrophotometer (Thermo Scientific™ Multiskan™ GO) at 570 nm using the absorbance at 690 nm as background.

### 4.4. Measurement of Apoptosis Markers Using Flow Cytometry

Cells were seeded and treated as described above in 24-well plates. After 24 h of treatment, the culture medium was discarded, the cells were washed twice with PBS, trypsinized and resuspended in complete growth medium. A suitable volume from the cell suspension, supplemented with PI dye (with 10 μg/mL final concentration), was used for the determination of viable cell numbers using the CytoFLEX™ S (Beckman Coulter™, Brea, CA, USA) flow cytometer. Then, cells were centrifuged at 300× *g* for 5 min and resuspended in HBSS (Hanks’ Balanced Salt Solution, Gibco™, 14185045). After determining viability and cell concentration, an appropriate amount of cell suspension (10^5^ cell/100 µL) was distributed onto a 96-well plate with the fluorescent probes in HBSS used for flow cytometry at the following manner. One drop of Annexin V Alexa Fluor ™ 488 Ready Flow Conjugate (Thermo Fisher Scientific, Invitrogen™, R37174) was added to a 0.5 mL cell suspension, containing 2.5 mM CaCl_2_, corresponding to 10^5^ cells or proportionately in smaller volumes and incubated for 10 min at room temperature. After 10 min, PI dye was also added to the suspension in a 10 μg/mL final concentration, and the samples were incubated for further 5 min in the dark at room temperature before being analyzed in a CytoFLEX™ S (Beckman Coulter™) flow cytometer. The emission of PI was measured on the ECD channel (610/20 nm), and the emission of the annexin V conjugate was measured on the FITC channel (525/40 nm). For determining caspase enzyme activity, the CellEvent™ Caspase-3/7 Green Detection Reagent (Invitrogen™, C10423) was used in a 5 µM final concentration. After the plate was incubated for 30 min at 37 °C in a cell culture incubator, the cells were analyzed in a CytoFLEX™ S (Beckman Coulter™) flow cytometer. The cell population was focused with unstained cells in both cases. Data were analyzed using FlowJo^®^ (version: 10.0.7r2); cell population was gated using unlabeled cells. Histograms are normalized to the samples’ mode.

### 4.5. Measurement of ROS and Lipid Peroxidation Using Flow Cytometry

Cells were seeded and treated as described above in 24-well plates. After the desired treatment time interval, the culture medium was discarded, the cells were washed twice with PBS, trypsinised, and resuspended in complete growth medium. Then cells were centrifuged at 300× *g* for 5 min and resuspended in HBSS. Cells were then distributed onto a 96-well plate with the fluorescent probes in HBSS used for flow cytometry at the following final concentrations: 5 µM dichlorofluorescein-diacetate (DCF-DA, Thermo Scientific™, D399), 2 µM BODIPY™ 581/591 C11 (Bodipy C11, Thermo Scientific™, D3861). After the plate was incubated for 30 min at 37 °C in a cell culture incubator, the cells were analyzed in a CytoFLEX™ S (Beckman Coulter™) flow cytometer. The cell population was focused with unstained cells, and all dyes were used separately to avoid interference. Data were analyzed using FlowJo^®^ (version: 10.0.7r2), cell population was gated using unlabeled cells. Histograms are normalized to the samples’ mode.

### 4.6. Measurement of GSH Levels

For the determination of cellular glutathione (GSH), monochlorbimane (mClB) derivatization followed by HPLC separation and fluorescent detection was used [44,45]. First, cells were trypsinized, resuspended in culture medium, centrifuged at 300× *g* for 5 min, and resuspended in TRIS buffer (20 mM, pH 8.0) in a 10^5^ cell/100 μL concentration; 100 μL was supplemented with 1 U/mL glutathione-S-transferase enzyme (GST) and mClB to reach a 1 mM final concentration. After a 15 min incubation in the dark at RT, the derivatization was stopped with the addition of 100% trichloroacetic acid (TCA) to reach a 10% final concentration. The solution was centrifuged at 15,000× *g* for 10 min at 4 °C, and the supernatant was used for GSH determination. For separation, a Waters Acquity UPLC H-Class system was used, equipped with an Acquity UPLC BEH C18 2.1 × 50 mm column with an average particle diameter of 1.7 µm. Gradient elution was used as 0.25% sodium-acetate (pH 3.5) and methanol. The detector was a Waters Acquity FLR fluorescent detector, with excitation and emission set to 395 and 477 nm, respectively. Quantitation was achieved by measuring GSH standards (Sigma-Aldrich^®^, G4251).

### 4.7. Isolation and Quantitation of Protein Samples

Cells were seeded in 6-well plates and treated as described above. After 24 h of treatment, cells were lysed in RIPA protein isolation buffer (150 mM NaCl, 1% NP-40, 50 mM Tris pH 8.0) supplemented with 1% protease inhibitor cocktail (Sigma^®^), 1% phosphatase inhibitor cocktail (Sigma^®^), and 1 mM PMSF. Samples were incubated on ice for 30 min then centrifuged at 14,000× *g* for 15 min at 4 °C. The supernatant was stored at −80 °C until protein analysis.

Protein samples were quantified using the Pierce™ BCA Protein Assay Kit (Thermo Scientific™, 23225), according to the manufacturer’s guidelines.

### 4.8. Analysis of Protein Samples Using Western Blot

SDS-PAGE was carried out via Cleaver Scientific omniPAGE. Proteins were transferred onto Millipore 0.45 µM nitrocellulose membrane. Immunoblotting was performed for non-phosphorylated proteins using TBS Tween (0.1%), containing 5% non-fat dry milk as blocking membrane, and 1% non-fat dry milk for antibody solutions, for phosphorylated proteins using TBS Tween (0.1%), containing 5% BSO as blocking membrane and for antibody solutions for phosphorylated proteins. Loading was controlled by developing membranes for GAPDH in each experiment.

The following primary antibodies were applied: Phospho-c-Jun (Ser63) (54B3) Rabbit mAb (Cell Signaling Technology^®^, Danvers, MA, USA, 2361), Anti-GAPDH (Santa Cruz Biotechnology, 6C5).

The following HRP-conjugated secondary antibodies were applied: HRP-Goat Anti-Rabbit IgG (Proteintech^®^, Rosemont, IL, USA, 00001-2), HRP-linked Anti-Mouse IgG (Proteintech^®^, 7076S).

The bands were visualized using Clarity™ ECL Western Blotting Substrate chemiluminescence detection kit (Bio-Rad, Hercules, CA, USA, 170-5060) and VWR™ (Radnor, PA, USA) Imager Chemi Premium gel documentation system with VWR™ Image Capture Software (version: 1.6.1.0). For densitometry analysis were carried out using ImageJ software bundled with 64-bit Java 1.8.0_172.

### 4.9. Statistical Analyses

All statistical analyses (One-way ANOVA or nonparametric Kruskal-Wallis ANOVA & Median Test) were carried out using TIBCO^®^ (Palo Alto, CA, USA) Statistica™ program (version: 14.4.0.0). *p* values were calculated with Dunnett’s test (after One-way ANOVA) or multiple comparisons (after Kruskal–Wallis test). Data are presented as average ± SD from at least three independent experiments.

## Data Availability

Not applicable.

## References

[1] Szarka A., Lőrincz T., Hajdinák P. (2022). Friend or Foe: The Relativity of (Anti)Oxidative Agents and Pathways. Int. J. Mol. Sci..

[2] Dolma S., Lessnick S.L., Hahn W.C., Stockwell B.R. (2003). Identification of Genotype-Selective Antitumor Agents Using Synthetic Lethal Chemical Screening in Engineered Human Tumor Cells. Cancer Cell.

[3] Yang W.S., Stockwell B.R. (2008). Synthetic Lethal Screening Identifies Compounds Activating Iron-Dependent, Nonapoptotic Cell Death in Oncogenic-RAS-Harboring Cancer Cells. Chem. Biol..

[4] Dixon S.J., Lemberg K.M., Lamprecht M.R., Skouta R., Zaitsev E.M., Gleason C.E., Patel D.N., Bauer A.J., Cantley A.M., Yang W.S. (2012). Ferroptosis: An Iron-Dependent Form of Nonapoptotic Cell Death. Cell.

[5] Gao M., Monian P., Pan Q., Zhang W., Xiang J., Jiang X. (2016). Ferroptosis Is an Autophagic Cell Death Process. Cell Res..

[6] Liu Y., He L., Liu B., Ying Y., Xu J., Yu M., Dang J., Liu K. (2022). Pharmacological Inhibition of Sphingolipid Synthesis Reduces Ferroptosis by Stimulating the HIF-1 Pathway. iScience.

[7] Alim I., Caulfield J.T., Chen Y., Swarup V., Geschwind D.H., Ivanova E., Seravalli J., Ai Y., Sansing L.H., Emma E.J. (2019). Selenium Drives a Transcriptional Adaptive Program to Block Ferroptosis and Treat Stroke. Cell.

[8] Bao W.D., Pang P., Zhou X.T., Hu F., Xiong W., Chen K., Wang J., Wang F., Xie D., Hu Y.Z. (2021). Loss of Ferroportin Induces Memory Impairment by Promoting Ferroptosis in Alzheimer’s Disease. Cell Death Differ..

[9] Mahoney-Sánchez L., Bouchaoui H., Ayton S., Devos D., Duce J.A., Devedjian J.C. (2021). Ferroptosis and Its Potential Role in the Physiopathology of Parkinson’s Disease. Prog. Neurobiol..

[10] Mi Y., Gao X., Xu H., Cui Y., Zhang Y., Gou X. (2019). The Emerging Roles of Ferroptosis in Huntington’s Disease. NeuroMol. Med..

[11] Hu C., Nydes M., Shanley K.L., Morales Pantoja I.E., Howard T.A., Bizzozero O.A. (2019). Reduced Expression of the Ferroptosis Inhibitor Glutathione Peroxidase-4 in Multiple Sclerosis and Experimental Autoimmune Encephalomyelitis. J. Neurochem..

[12] Zhang J., Wang B., Yuan S., He Q., Jin J. (2022). The Role of Ferroptosis in Acute Kidney Injury. Front. Mol. Biosci..

[13] Lőrincz T., Jemnitz K., Kardon T., Mandl J., Szarka A. (2015). Ferroptosis Is Involved in Acetaminophen Induced Cell Death. Pathol. Oncol. Res..

[14] Szarka A., Kapuy O., Lőrincz T., Bánhegyi G. (2021). Vitamin C and Cell Death. Antioxid. Redox Signal..

[15] McCubrey J.A., LaHair M.M., Franklin R.A. (2006). Reactive Oxygen Species-Induced Activation of the MAP Kinase Signaling Pathways. Antioxid. Redox Signal..

[16] Shen H.M., Liu Z.G. (2006). JNK Signaling Pathway Is a Key Modulator in Cell Death Mediated by Reactive Oxygen and Nitrogen Species. Free Radic. Biol. Med..

[17] Shrivastava A., Aggarwal B.B. (1999). Antioxidants Differentially Regulate Activation of Nuclear Factor-ΚB, Activator Protein-1, c-Jun Amino-Terminal Kinases, and Apoptosis Induced by Tumor Necrosis Factor: Evidence That JNK and NF-ΚB Activation Are Not Linked to Apoptosis. Antioxid. Redox Signal..

[18] Ueda S., Masutani H., Nakamura H., Tanaka T., Ueno M., Yodoi J. (2002). Redox Control of Cell Death. Antioxid. Redox Signal..

[19] Cross T.G., Scheel-Toellner D., Henriquez N.V., Deacon E., Salmon M., Lord J.M. (2000). Serine/Threonine Protein Kinases and Apoptosis. Exp. Cell Res..

[20] Lőrincz T., Holczer M., Kapuy O., Szarka A. (2019). The Interrelationship of Pharmacologic Ascorbate Induced Cell Death and Ferroptosis. Pathol. Oncol. Res..

[21] Hibi M., Lin A., Smeal T., Minden A., Karin M. (1993). Identification of an Oncoprotein- and UV-Responsive Protein Kinase That Binds and Potentiates the c-Jun Activation Domain. Genes Dev..

[22] Dérijard B., Hibi M., Wu I.H., Barrett T., Su B., Deng T., Karin M., Davis R.J. (1994). JNK1: A Protein Kinase Stimulated by UV Light and Ha-Ras That Binds and Phosphorylates the c-Jun Activation Domain. Cell.

[23] Plattner R., Gupta S., Khosravi-Far R., Sato K.Y., Perucho M., Der C.J., Stanbridge E.J. (1999). Differential Contribution of the ERK and JNK Mitogen-Activated Protein Kinase Cascades to Ras Transformation of HT1080 Fibrosarcoma and DLD-1 Colon Carcinoma Cells. Oncogene.

[24] Yang W.S., Sriramaratnam R., Welsch M.E., Shimada K., Skouta R., Viswanathan V.S., Cheah J.H., Clemons P.A., Shamji A.F., Clish C.B. (2014). Regulation of Ferroptotic Cancer Cell Death by GPX4. Cell.

[25] de Souza I., Ramalho M.C.C., Guedes C.B., Osawa I.Y.A., Monteiro L.K.S., Gomes L.R., Rocha C.R.R. (2022). Ferroptosis Modulation: Potential Therapeutic Target for Glioblastoma Treatment. Int. J. Mol. Sci..

[26] Friedmann Angeli J.P., Schneider M., Proneth B., Tyurina Y.Y., Tyurin V.A., Hammond V.J., Herbach N., Aichler M., Walch A., Eggenhofer E. (2014). Inactivation of the Ferroptosis Regulator Gpx4 Triggers Acute Renal Failure in Mice. Nat. Cell Biol..

[27] Bruckner S.R., Estus S. (2002). JNK3 Contributes to C-Jun Induction and Apoptosis in 4-Hydroxynonenal-Treated Sympathetic Neurons. J. Neurosci. Res..

[28] Parola M., Robino G., Marra F., Pinzani M., Bellomo G., Leonarduzzi G., Chiarugi P., Camandola S., Poli G., Waeg G. (1998). HNE Interacts Directly with JNK Isoforms in Human Hepatic Stellate Cells. J. Clin. Investig..

[29] Yin H., Xu L., Porter N.A. (2011). Free Radical Lipid Peroxidation: Mechanisms and Analysis. Chem. Rev..

[30] Dixon S.J., Patel D.N., Welsch M., Skouta R., Lee E.D., Hayano M., Thomas A.G., Gleason C.E., Tatonetti N.P., Slusher B.S. (2014). Pharmacological Inhibition of Cystine–Glutamate Exchange Induces Endoplasmic Reticulum Stress and Ferroptosis. eLife.

[31] Hajdinák P., Czobor Á., Szarka A. (2019). The Potential Role of Acrolein in Plant Ferroptosis-like Cell Death. PLoS ONE.

[32] Singh R., Wang Y., Schattenberg J.M., Xiang Y., Czaja M.J. (2009). Chronic Oxidative Stress Sensitizes Hepatocytes to Death from 4-Hydroxynonenal by JNK/c-Jun Overactivation. Am. J. Physiol. Liver Physiol..

[33] Czaja M.J., Liu H., Wang Y. (2003). Oxidant-Induced Hepatocyte Injury from Menadione Is Regulated by ERK and AP-1 Signaling. Hepatology.

[34] Liu H., Lo C.R., Czaja M.J. (2002). NF-ΚB Inhibition Sensitizes Hepatocytes to TNF-Induced Apoptosis through a Sustained Activation of JNK and c-Jun. Hepatology.

[35] Malhi H., Bronk S.F., Werneburg N.W., Gores G.J. (2006). Free Fatty Acids Induce JNK-Dependent Hepatocyte Lipoapoptosis. J. Biol. Chem..

[36] Schwabe R.F., Uchinami H., Qian T., Bennett B.L., Lemasters J.J., Brenner D.A. (2004). Differential Requirement for C-Jun NH2-Terminal Kinase in TNFalpha- and Fas-Mediated Apoptosis in Hepatocytes. FASEB J..

[37] Okada M., Shibuya K., Sato A., Seino S., Suzuki S., Seino M., Kitanaka C. (2014). Targeting the K-Ras-JNK Axis Eliminates Cancer Stem-like Cells and Prevents Pancreatic Tumor Formation. Oncotarget.

[38] Céspedes M.V., Sancho F.J., Guerrero S., Parreño M., Casanova I., Pavón M.A., Marcuello E., Trias M., Cascante M., Capellà G. (2006). K-Ras Asp12 Mutant Neither Interacts with Raf, nor Signals through Erk and Is Less Tumorigenic than K-Ras Val12. Carcinogenesis.

[39] Zhou Y., Rideout W.M., Zi T., Bressel A., Reddypalli S., Rancourt R., Woo J.K., Horner J.W., Chin L., Chiu M.I. (2010). Chimeric Mouse Tumor Models Reveal Differences in Pathway Activation between ERBB Family-and KRAS-Dependent Lung Adenocarcinomas. Nat. Biotechnol..

[40] Collins M.A., Pasca di Magliano M. (2014). Kras as a Key Oncogene and Therapeutic Target in Pancreatic Cancer. Front. Physiol..

[41] Dhanasekaran D.N., Premkumar Reddy E. (2017). JNK-Signaling: A Multiplexing Hub in Programmed Cell Death. Genes Cancer.

[42] Bryan H.K., Olayanju A., Goldring C.E., Park B.K. (2013). The Nrf2 Cell Defence Pathway: Keap1-Dependent and -Independent Mechanisms of Regulation. Biochem. Pharmacol..

[43] Nishizawa H., Yamanaka M., Igarashi K. (2022). Ferroptosis: Regulation by Competition between NRF2 and BACH1 and Propagation of the Death Signal. FEBS J..

[44] Hajdinák P., Czobor Á., Lőrincz T., Szarka A. (2018). The Problem of Glutathione Determination: A Comparative Study on the Measurement of Glutathione from Plant Cells. Period. Polytech. Chem. Eng..

[45] Lőrincz T., Szarka A. (2017). The Determination of Hepatic Glutathione at Tissue and Subcellular Level. J. Pharmacol. Toxicol. Methods.

